# Evaluation of high-risk Human papillomaviruses type distribution in cervical cancer in Sichuan province of China

**DOI:** 10.1186/1471-2407-8-202

**Published:** 2008-07-22

**Authors:** En-qi Wu, Guo-nan Zhang, Xiang-hui Yu, Yuan Ren, Ying Fan, Yong-ge Wu, Wei Kong, Xiao Zha

**Affiliations:** 1 College of Life Science, Jilin University, Changchun 130012, PR China; 2 Department of Molecular Pharmacology, Sichuan Tumor Hospital, Chengdu 610041, PR China; 3 Department of Gynecologic Oncology, Sichuan Tumor Hospital, Chengdu 610041, PR China

## Abstract

**Background:**

Infection with high-risk human papillomavirus is an important factor associated with cervical cancer, and the distribution of HPV types varies greatly worldwide. Determination of type-specific HPV prevalence constitutes an important step towards the development of vaccines for the prevention of cervical cancer.

**Methods:**

The human papillomavirus (HPV) genotypes in 190 cervical cancer specimens taken from the Sichuan province, the most populous province of Southwest China, were detected by a combination of MY09/11 consensus primers PCR (MY09/11 PCR), type-specific primers one-step PCR (One-step TS PCR) and E6/E7 gene type-specific primers nested PCR (Nested TS PCR). The prevalence and distribution of HPV in patients with cervical cancer, especially for HPV types 16, 18, 52, 58 and 59, suspected to be most common in certain parts of China, was investigated.

**Results:**

The HPV infection rates detected by MY09/11 PCR, One-step TS PCR and Nested TS PCR were 159 (83.7%), 145 (76.3%) and 172 (90.5%), respectively. The overall HPV prevalence was 93.2% (177/190). The positive specimens for HPV16, 18, 52, 58 and 59 detected by One-step TS-PCR were 111 (58.4%), 14 (7.4%), 6 (3.2%), 13 (6.8%) and 4 (2.1%), respectively. By Nested TS-PCR analysis, the detection rates of HPV16, 52, 58 and 59 were increased to 140 (73.7%), 30 (15.8%), 37 (19.5%) and 25 (13.2%), while only 4 (2.1%) additional specimens were found to be infected with HPV18.

**Conclusion:**

Our data demonstrate that, besides HPV 16, which was found to be the most prevalent type, HPV types 58, 52 and 59 are more prevalent than HPV18 in women with cervical cancer in the Sichuan area of China.

## Background

Cervical cancer is the second most common cancer in women; each year approximately 500,000 women worldwide are diagnosed with invasive cervical cancer and more than half of them die of this disease. Eighty percent of these deaths occur in developing countries. In China, there is an annual incidence of about 46,000 cases, and cervical cancer presents a major health problem [1]. It is widely believed that persistent infection with high-risk human papillomavirus (HPV) represents the prime risk factor in cervical carcinogenesis [2,3]. More than 100 different genotypes of HPV have been identified thus far, and there are approximately 40 known genital HPVs. At least 18 of these viruses have been associated with cervical cancer [4,5]. Since the distribution of HPV genotypes in various geographical areas and populations varies widely [6-8], it is important to delineate the prevalence of different HPV types found in cervical cancer for guiding the selection of vaccine candidates and studying the oncogenic function of each genotype.

The most widely used method for HPV detection in cervical cancers is based on polymerase chain reaction (PCR) using either consensus or type-specific primers for the amplification of HPV DNA. The type-specific PCR primers have high specificity and sensitivity in the detection of certain HPV types, and the consensus PCR primers can detect a broad spectrum of HPV types in each reaction. Several investigators have introduced the nested PCR method for detection of HPV infection, using both consensus (MY09/MY11, GP5+/GP6+) and type-specific primers, and it has been found that the nested PCR assay is an extremely sensitive and reliable means for HPV detection because the viral DNA can be amplified with extremely low copy number [9-11].

It is well known that HPV16 and HPV18 are the most predominant high-risk types found in cervical cancer tissue in countries around the world, while the prevalence of the other high-risk HPV types varies from one region to another [8]. The prevalence and type distribution of HPV types in cervical cancer in China is still uncertain. Huang et al. [12] found that HPV52 and 58 were prevalent in cervical cancers from Chinese women living in Shanghai, while Liu et al. [13] reported that HPV58 and 59 were found to be the third most common genotypes infected in women with cervical cancer from Guangzhou and Changsha, following HPV16 and 18. In addition, a multi-center study of HPV infection in cervical cancer including 5 geographical regions of China reported that HPV16, 18, 52 and 58 were the most common genotypes in China [14]. These findings indicate that HPV types 16, 18, 52, 58 and 59 should be included in the evaluation of HPV detection assays when studying Chinese women.

In this study, MY09/11 PCR and One-step TS PCR assays were performed to evaluate the prevalence and distribution of high-risk HPV types. To describe the infections of HPV types 16, 18, 52, 58 and 59 in patients with cervical cancer in more detail, Nested TS PCR assays were performed for 190 cervical cancer specimens from the Sichuan province, the most populous province of southwest China, which covers an area of 480,000 square kilometers and has a population of about 90 million people.

## Methods

### Specimen collection

The present study was carried out with the approval of the ethical committee of Sichuan Tumor Hospital, and patient consent was obtained for the collection of cervical cancer tissues. One hundred and ninety fresh cervical cancer tissues were obtained via surgical operation from patients from the Sichuan province between October 2004 and October 2006 and preserved at -70°C. The patients ranged in age from 17 to 71 years, with a mean of 46 years. Of the 190 tumor specimens, 176 were diagnosed as squamous-cell carcinoma (SCC), 12 as adenocarcinoma (AC) and 2 as SCC\AC mixed tumors.

### DNA extraction

DNA was extracted from frozen tissue specimens by DNeasy Tissue Kit (QIAGEN), according to the manufacturer's instructions. Extracted DNA was eluted with 100 μl AE buffer (10 mM Tris, pH 8.5) and stored at -20°C until it was analyzed. The quality of extracted DNA was checked by PCR amplification of β-globin gene (the primer forward 5'-CAACTTCATCCACGTTCACC-3' and reverse 5'-GAAGAGCCAAGGACAGGTAC-3') [15].

### MY09/11 PCR assay

MY09/11 PCR was performed with all specimens in order to detect a broad spectrum of HPV genotypes, as described previously [16]. The amplified DNA fragments were identified by electrophoresis in 1.5% agarose gel with ethidium bromide.

### One-step TS PCR assay

All specimens were tested by One-step TS PCR assays using type-specific primers for HPV types 16, 18, 52, 58 and 59, which were previously described by Sotlar et al.[17] (Table 1). The PCR was performed in 50 μl of reaction mixture containing 1×PCR buffer, 2.5 mmol/L MgCl_2_, 200 μmol/L of each deoxynucleoside, 0.5 μmol/L of sense and anti-sense primer, 5 μl of template DNA and 1U Taq DNA polymerase (MBI, Fermentas) by a 35 cycle protocol: denaturation at 94°C for 1 min, annealing at 56°C for 1 min and extension at 72°C for 1 min, with an initial denaturation at 94°C for 5 min and a final extension at 72°C for 5 min.

**Table 1:** Primers used for the detection of HPV DNA in cervical cancer tissues

Name		Target DNA	Nucleotide sequence(5'-3')	Prime position	Product size(bp)
Primers for the One-step TS PCR and the second round of the Nested TS PCR*					

1601	Sense	HPV16 E6 to E7 gene	CACAGTTATGCACAGAGCTGC	141–161	457
1602	Antisense		CATATATTCATGCAATGTAGGTGTA	597–573	
1801	Sense	HPV18 E6 gene	CACTTCACTGCAAGACATAGA	170–-190	322
1802	Antisense		GTTGTGAAATCGTCGTTTTTCA	491–470	
5201	Sense	HPV52 E6 gene	TAAGGCTGCAGTGTGTGCAG	178–197	229
5202	Antisense		CTAATAGTTATTTCACTTAATGGT	406–383	
5801	Sense	HPV58 E6 gene	GTAAAGTGTGCTTACGATTGC	297–317	274
5802	Antisense		GTTGTTACAGGTTACACTTGT	570–550	
5901	Sense	HPV59 E6 gene	CAAAGGGGAACTGCAAGAAAG	159–179	215
5902	Antisense		TATAACAGCGTATCAGCAGC	373–354	

Primers for the first round amplifications of Nested TS PCR**					

LF16	Sense	HPV 16 URR to E7 gene	AGGGCGTAACCGAAATCGGT	27–45	632
LR16	Antisense		CTGAGCTGTCATTTAATTGCTCA	658–636	
LF18	Sense	HPV 18 URR to E7 gene	GGGAGTAACCGAAAACGGT	35–53	662
LR18	Antisense		TCCTCTGAGTCGCTTAATTGCTC	696–674	
LF52	Sense	HPV 52 URR to E7 gene	GGGTGTAACCGAAAACGGT	31–49	619
LR52	Antisense		CTGAGCTGTCACCTAATTGCTCA	649–627	
LF58	Sense	HPV58 URR to E7 gene	GGGTGTAACCGAAAACGGT	33–51	638
LR58	Antisense		CTGAGCTGTCACATAATTGCTCA	670–648	
LF59	Sense	HPV59 URR to E7 gene	GTTAAGACCGAAAACGGTG	1–19	648
LR59	Antisense		GAGTCGGAGTCAGGTAATTGCTC	648–626	

* designed by Sotlar et al.[17]; **Designed specifically for this study.

### Nested TS PCR assay

To increase the sensitivity of the detection for HPV16, 18, 52, 58 and 59, the specimens negative by the One-step TS PCR for any genotype were further amplified by Nested TS PCR. The TS primers for HPV types 16, 18, 52, 58 and 59 of the first round of amplifications were designed specifically for this study to amplify a wider region (raging 619 to 662 bp) than those targeted by primers used in the One-step TS PCR assay, which were used in the second round of amplification of the Nested TS PCR (Table 1).

The first round reaction of nested PCR was performed under the following conditions: 40 cycles at 94°C for 1 min, 50°C for 1 min, and 72°C for 1 min. The first cycle was preceded by a 3 min denaturation step and the last cycle was followed by a 10 min extension step. The second round reaction of nested PCR was performed in 25 μl of reaction mixture, using 2 μl of PCR product from the first round reactions as template DNA. The reaction mixture contained 1×PCR buffer, 2.5 mmol/L MgCl_2_, 0.5 μmol/L of sense and anti-sense primer, 200 μmol/L of each deoxynucleoside, and 1 U Taq DNA polymerase (MBI, Fermentas). Specimens were amplified through 40 cycles at 95°C for 1 min, 55°C for 1 min, and 72°C for 1 min. This was followed by a final extension at 72°C for 5 min.

Five specimens, positive for HPV 16, 18, 52, 58 and 59 (as detected by One-step TS PCR and confirmed by sequencing), were used as positive controls. The reaction mixture, containing no DNA, was the negative control in PCR. When the nested PCR was performed, the product of the negative control in the first round was used as template in nested PCR amplification as negative controls to detect any contamination. The amplified DNA fragments were identified by electrophoresis in 1.5% agarose gel with ethidium bromide.

### DNA Sequencing

The MY09/11 PCR positive fractions of specimens which were negative for HPV16, 18, 52, 58 and 59 by One-step TS PCR assay were purified by PCR purification (QIAGEN) and sequenced by using BigDye Terminator v3.1 Cycle Sequencing Kit (PE Applied Biosystems) on an ABI 3730 Genetic Analyzer (PE Applied Biosystems). The HPV genotype was identified by comparing the sequence with that reported in GenBank using the BLAST 2.0 software server .

### Statistical analysis

McNemar's tests were performed to analyze statistical significance of the detection frequency data. Statistical analyses were performed with Statistical Analysis System software package (SPSS 13.0, SPSS, Chicago, IL).

## Results

The results of HPV infection detection in the 190 cervical cancer tissue specimens analyzed by MY09/11 PCR and type-specific primer mediated one-step and nested PCR assays are shown in Table 2.

**Table 2:** HPV detection by the MY09/11 PCR, the One-step TS PCR and the Nested TS PCR in cervical cancer specimens (N = 190).

	MY09/11 PCR	One-step TS PCR	One-step TS PCR + Nested TS PCR*	Total
Positive	159	145	172	177
Negative	31	45	18	13
Positive rate (%)	83.7	76.3	90.5	93.2

*Combination of One-step TS PCR and Nested TS PCR.

Detected by One-step TS PCR assay, the percentage of positive specimens for HPV16, 18, 52, 58 and 59 was 111 (58.4%), 14 (7.4%), 6 (3.2%), 13 (6.8%) and 4 (2.1%), respectively. Overall, 76.3% (145/190) of the specimens were positive for one of these five HPV types. Using MY09/11 PCR assay, the HPV positive rate was 83.7% (159/190). These results are compared in Table 3. Twenty of the 159 positive specimens detected by MY09/11 PCR assays were negative for DNA from any of the five HPV genotypes in the One-step TS PCR assay. Among them, 14 cases were successfully typed by direct DNA sequencing, including 5 (2.6%) cases of HPV45, 4 (2.1%) cases of HPV31, 2 (1.1%) cases each of HPV33 and 39, and 1 (0.5%) case of HPV82; 6 specimens were defined as containing unknown type HPV infection, due to poor sequencing results.

**Table 3:** Comparison of detection of HPV by E6 One-step TS PCR assay with detection by MY09/11 PCR assays for 190 specimens.

		MY09/11 PCR		
		Positive (%)	Negative (%)	Total (%)

One-step TS PCR	Positive (%)	139 (73.2)	6 (3.1)	145 (76.3)
	Negative (%)	20 (10.5)	25 (13.2)	45 (23.7)
	Total (%)	159 (83.7)	31 (16.3)	190

**Figure 1:**
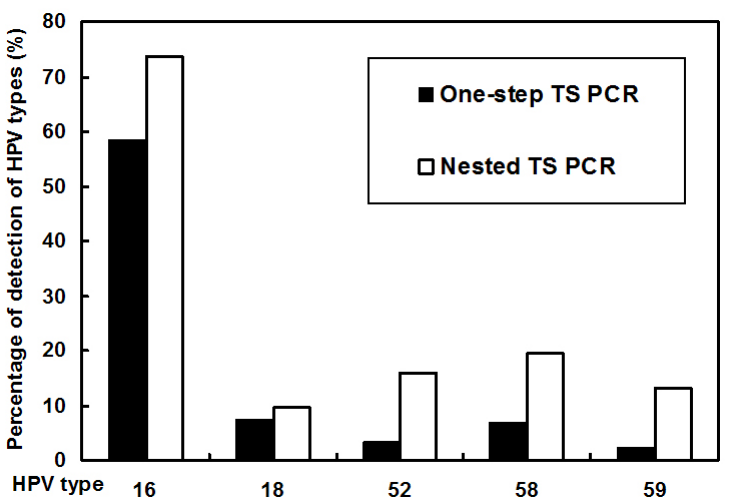
Frequency of detection of HPV types 16, 18, 52, 58 and 59 in 190 samples.

Detected by Nested TS PCR analysis, 29 of 79, 4 of 176, 24 of 184, 24 of 177 and 21 of 186 specimens that were negative from the One-step TS PCR were identified as positive for HPV16, 18, 52, 58 and 59, respectively. The positive rates of HPV16, HPV52, HPV58 and HPV59 were increased significantly (from 58.4% to 73.7% for HPV16, P < 0.001; from 3.2% to 15.8% for HPV52, P < 0.001; from 6.8% to 19.5% for HPV58, P < 0.001 and from 2.1% to 13.2% for HPV59, P < 0.001), while the HPV18 positive rate was increased from 7.4% to 9.5% (p = 0.125), only 2.1% higher (Fig 1). The positive rate of specimens infected with these five HPV types was increased to 90.5% (172/190) in all specimens.

Table 4 shows the number of single and multiple infections detected by a combination of the three aforementioned PCR methods. Of the 190 specimens, 101 (53.2%) were identified as infected with a single HPV type and 76 (40.0%) with multiple HPV types.

**Table 4:** Number and proportion of single and multiple infections in 190 specimens.

Single infection		Dual infection		Triple infection	
HPV types	Case (%)	HPV types	Case (%)	HPV types	Case (%)

16	79 (41.6)	16/18	5 (2.6)	16/18/58	2 (1.1)
18	6 (3.2)	16/31	1 (0.5)	16/18/59	1 (0.5)
31	2 (1.1)	16/33	1 (0.5)	16/52/58	4 (2.1)
45	2 (1.1)	16/39	1 (0.5)	16/58/59	1 (0.5)
52	4 (2.1)	16/45	1 (0.5)	16/52/59	2 (1.1)
58	5 (2.6)	16/52	11 (5.8)	16/33/59	1 (0.5)
59	2 (1.1)	16/58	19 (10.0)		
82	1 (0.5)	16/59	11 (5.8)		
		18/31	1 (0.5)		
		18/52	1 (0.5)		
		18/59	2 (1.1)		
		39/52	1 (0.5)		
		45/52	1 (0.5)		
		45/58	1 (0.5)		
		52/58	3 (1.6)		
		58/59	2 (1.1)		
		52/59	3 (1.6)		

Total	101 (53.2)	Total	65 (34.2)	Total	11 (5.8)

## Discussion

The overall HPV positive rate detected by a combination of MY09/11 PCR and One-step TS PCR was 86.8% (165/190), which is close to a meta-analysis of HPV prevalence among 5954 ICC cases in Asia (85.9%) [18] and slightly higher than the rate reported in a meta-analysis in China alone (83.7%) [19]. HPV16 was the most common HPV type in this area, accounting for 58.4% of all cases, similar to the results of the meta-analysis in China (58.7%) [19]. HPV18, with a detection rate (7.4%) lower than that reported in Asia (14.9%) [18], was the second most common type, followed by 58 (6.8%), 52 (3.2%), 45 (2.6%), 59 (2.1%), 31 (2.1%), 39 (1.1%), 33 (1.1%) and 82 (0.5%). Six (3.2%) specimens remained untyped, and three specimens were found to be infected by multiple types of HPV, including combinations of HPV16/HPV18, HPV16/HPV52 and HPV16/HPV59. The HPV positive rate detected by MY09/11 PCR (83.7%) was significantly higher (p = 0.009) than that detected by one-step TS PCR (76.3%). There were 20 specimens that were positive for HPV when using MY09/11 PCR but not One-step TS PCR as well as 6 specimens that had the reverse results. This discrepancy reflects the fact that these two methods vary in their abilities to detect HPV. The One-step TS PCR can determine specific HPV types due to its high specificity; however, this method requires a special pair of primers for detecting each type of HPV, and it is difficult to cover all HPV types that could be found in precancer and cervical cancer [6-8]. In this study, the One-step TS PCR assay was used only for five high-risk HPV types: 16, 18, 52, 58 and 59. Consensus primer PCR made it possible to detect various virus types simultaneously; however, because the amplified sequence of consensus primers is usually located in the L1 gene, which might be disrupted frequently as a consequence of HPV integration [20], the consensus primer mediated PCR method is not always suitable for detecting the integrated HPV in some cases. Therefore, consensus primer PCR has suboptimal sensitivity compared with the type-specific primer PCR methods.

The sensitivity and specificity of various HPV detection methods suggest a reason for the variance among analyses of the prevalence of HPV in cervical cancer in China. In order to characterize infections by HPV types 16, 18, 52, 58 and 59 in patients with cervical cancer in more detail, a reliable and sensitive detection system is necessary. Sotlar et al.[17] have described a nested multiplex PCR (NMPCR) assay that combines degenerate E6/E7 consensus primers and type-specific primers for the detection and typing of most high risk human papillomavirus genotypes, and they suggest that the NMPCR method is a sensitive and useful tool for HPV DNA detection, especially when exact HPV genotyping and the identification of multiple HPV infections are required. However, in a pre-experiment using NMPCR, this method failed to detect the presence of multiple infections that identified by the One-step TS PCR method. These multi-infections were detectable if primers specific to only one HPV genotype were included in the second step of NMPCR (data not shown). This may be due in part to the fact that the multi-primers employed in the second step reaction of NMPCR included excessive off-target primers which caused considerable primer-dimer formation within pairs of primers or inefficient amplification [21]. Therefore, the TS primers for HPV type 16, 18, 52, 58 and 59 for the first round of amplification of the Nested TS PCR assay were specifically designed for this study.

**Figure 2:**
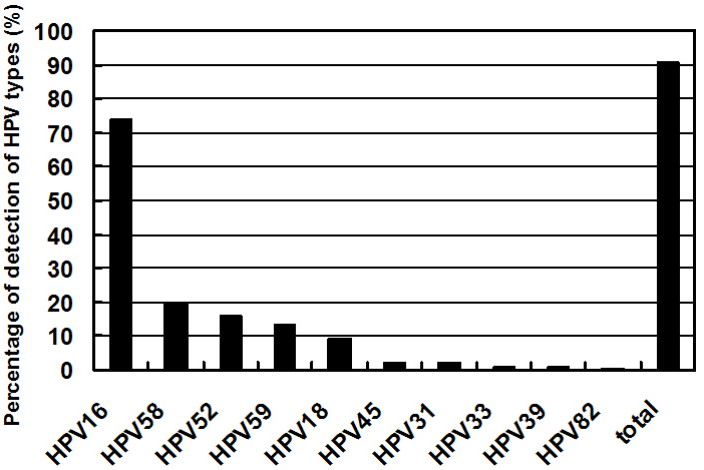
Distribution of HPV types in 190 patients with cervical cancer from Sichuan province.

Combination of one-step TS PCR and Nested TS PCR increased the positive rate of HPV types 16, 52, 58 and 59 greatly and identified many more multiple infections. Multiple HPV infections in women with cervical pathology have been reported widely and the detection rates vary drastically, depending on methods used, from 0% to 40% [22]. In this study, 40% of the specimens were identified as being infected with multiple HPV types. Note that most specimens infected with multiple types of HPV contain at least one or two high-risk HPV types that can be detected by One-step TS PCR. The copy numbers of the HPV type detected by One-step TS PCR or MY 09/11 PCR should be much higher than those detected by Nested TS PCR, which are more likely to be clonally related to the tumor. Although in multiple HPV infection cases, the roles of different high risk HPV types and different viral loads in development of cervical cancer are remain unclear, the Nested TS PCR assay identifies which HPV types are more prevalent than others in a geographical area and gives more information about HPV type distribution in cervical cancer. Combining the results of these three methods, the overall HPV positive rate was 93.2% (177/190). The order of infection rate of these five HPV types in the Sichuan area changed after the Nested TS PCR analysis. The distributions of HPV types detected by the combination of the three methods are shown in Fig. 2. HPV16 is still the most common HPV type in this area, but HPV58, not HPV18, is the second most common HPV type followed by HPV52, 59, and then types 18, 45, 31, 33, 39 and 82. This indicates that HPV58, 52 and 59 cause more infections than HPV18 in cervical cancer patients from the Sichuan province. Notably, there is a high prevalence of HPV52, 58 and 59 in cervical cancers in China. These data will have implications on the design of HPV screening systems and the development of HPV vaccines in China.

## Conclusion

In this study, Nested TS PCR assays were performed to supplement One-step TS PCR and MY09/11 PCR assays for investigating the distribution of HPV genotypes in 190 cervical cancer specimens from China. The results demonstrate that improved sensitivity of the detection methods allows for increased findings of HPV16, 52, 58 and 59, but not HPV18. HPV58, 52 and 59 infections seem to be more prevalent than HPV18 in women with cervical cancer from the Sichuan area of China. The use of Nested TS PCR assays for detecting HPV16, 18, 52, 58 and 59 and for detecting the prevalence of other HPV genotypes deserves further investigation.

## Competing interests

The authors declare that they have no competing interests.

## Authors' contributions

EW carried out the PCR analysis, participated in the writing of the manuscript. GZ performed the clinical diagnoses and surgical operations, participated in data collection and statistical analysis. XY participated in the discussion and implementation of the methods and the writing of manuscript. YR carried out the genomic DNA extraction. YF participated in the surgical operations and collected the tissue specimens. YW carried out the sequencing analysis. WK and XZ conceived the study, participated in its design and prepared the manuscript draft for submission. All authors read and approved the final manuscript.

## Pre-publication history

The pre-publication history for this paper can be accessed here:


